# Impact of the COVID-19 Pandemic on Anaesthesiology and Reanimation Residency Training in Turkey

**DOI:** 10.5152/TJAR.2022.21320

**Published:** 2022-04-01

**Authors:** Bahar Sakızcı Uyar, Alp Alptekin, Aslı Dönmez

**Affiliations:** 1 Department of Anaesthesiology and Reanimation, University of Health Sciences Dışkapı Yıldırım Beyazıt Training and Research Hospital, Ankara, Turkey

**Keywords:** COVID-19, pandemic, anaesthesiology and reanimation, resident education, training, impact

## Abstract

**Objective:**

The purpose of this study was to evaluate the impact of the pandemic on clinical practice and education of anaesthesiology and reanimation residents in Turkey.

**Methods:**

A 33-question web-based survey was sent to anaesthesiology and reanimation residents in Turkey. Residents were asked about their clinical practice and education before and during the pandemic and personal perspectives on working conditions and training.

**Results:**

A total of 223 residents participated. Median working time in the intensive care unit of 2.5 months/year before the pandemic increased to 6 months/year during the pandemic (*P* < .001). Median working time in the operating room of 9 months/year decreased to 6 months/year (*P* < .001). The time working in the algology and anaesthesiology outpatient clinic decreased significantly (both *P* < .001). Neuraxial and peripheral nerve block practices decreased (*P* = .002 and *P* = .023, respectively). The number of night shifts per month increased (*P* < .001). While the average number of beds in intensive care units was 14, it increased to 19.5 beds (*P* < .001). The education time for lecture and clinical case discussion decreased (*P* < .001), but medical meeting attendance did not change (*P* = .174). Eighty-seven percent of the residents reported that night shifts were very intense and intense during the pandemic. For 87.3% of the respondents, the workload increased, and 71.6% of the respondents reported a decrease in theoretical education and 66.7% in practical training. Sixty-three percent of last year residents reported that they were negatively and very negatively affected in making the thesis.

**Conclusion:**

The pandemic had a negative impact on anaesthesiology and reanimation residency training programs in Turkey.

## Main Points

The pandemic had a negative impact on anaesthesiology and reanimation residency training programs in Turkey. 

The working time of anaesthesiology and reanimation residents in intensive care units increased and the working conditions became intense during the pandemic in Turkey. 

However, anaesthesiology and algology practices decreased.

## Introduction

With the declaration of coronavirus disease 19 (COVID-19) as a pandemic, healthcare systems have regulated their systems aiming to reduce the spread of the infection and use resources efficiently.^1^ Elective surgeries and clinic visits have been canceled in many countries. Physicians have used telemedicine for clinical assessment to facilitate social distancing.^2,3^ Anaesthesiology and reanimation specialists and residents, on the other hand, have provided frontline care by staffing expanded intensive care units (ICU).^4^

Intensive working conditions at hospitals, social distancing, and in some countries, total lockdown have also impacted the resident’s education. Face-to-face medical meetings, courses, and exams have been canceled. On the other hand, there has been an opportunity for learning from seniors worldwide via numerous free webinars.^4^ However, anaesthesia is a hands-on specialty, and therefore, online learning may not be enough.^5^

This study aimed to evaluate the impact of the pandemic on clinical practice and education of anaesthesiology and reanimation residents in Turkey by conducting a web-based survey. To the best of our knowledge, this is the first nationwide study evaluating the clinical practice and education of anaesthesiology and reanimation residents during the COVID-19 pandemic. 

## Methods

Ethics committee approval (105/07, February 22, 2021) and Republic of Turkey Ministry of Health approval (2021-01-04T13_56_45) were obtained for this nationwide cross-sectional study. A 33-question, web-based survey was created on the Google Form platform by all authors (https://docs.google.com/forms/d/14k7wBGStmG3gKeRoF9edA9dpZBvREoWBKq-hM-n_uzg/edit). A pilot study was conducted with a small sample of anaesthesiology and reanimation residents (n = 10) to validate the content, clarity of questions, and time required to complete the survey. The link to the survey was mailed to anaesthesiology and reanimation residents in Turkey by contacting the Turkish Society of Anaesthesiology and Reanimation Specialists. The participation was voluntary and there was a statement in the survey description as: “You accept your data will be processed by continuing.” The survey comprised 24 open-ended, 7 multiple-choice, and 3 Likert scale questions grouped into 3 parts. The first part collected demographic data. In the second part, residents were asked about clinical practice and education before and during the pandemic. The third part consisted of questions about the personal perspectives of the residents on working conditions and education. Answering all questions except the last 2 was mandatory. Question 32 addressed only to residents from the last year of the training program. The last question asked open-ended anything they wanted to add on the topic. The survey was accessible from April 06, 2021, to May 11, 2021. 

### Statistical Analysis

Statistical analysis were performed using the Statistical Package for the Social Sciences for Windows version 20.0 (IBM Corp., Armonk, NY, USA). Data are shown as numbers (percentage) or medians, ranges, and quartiles. The normality of the distributions was analyzed using the “Kolmogorov–Smirnov” and “Shapiro–Wilk” tests. Since none of the numerical data showed normal distribution, the Wilcoxon test was used before and during pandemic analyses. Categorical variables were analyzed using the chi-square test or Fisher’s exact test, as appropriate. Data were analyzed at a 95% CI level. *P* value less than .05 was considered as statistically significant.

## Results

Among 1070 anaesthesiology and reanimation residents registered in the database of the Turkish Society of Anaesthesiology and Reanimation Specialists, 223 (20.8%) participated in the survey, and 119 of the participants (53.4%) were female and 104 (43.6%) were male. Residents’ age ranged from 24 to 49 with a median of 29, and 65 of the participants (29.1%) were employed in training and research hospitals and 158 (70.9%) in university. All the participants worked in COVID-19 reference hospitals. Regarding the year of residency, 8.5% (n = 19) were in the first year, 16.1% (n = 36) in the second year, 23.8% (n = 53) in the third year, 18.4% (n = 41) in the fourth year, and 33.2% (n = 74) in the fifth (last) year (Table 1).

The analyses other than demographic data were made after excluding the first-year residents since the first-year residents answered the open-ended questions regarding before the pandemic as “I did not work.” Table 2 details the responses regarding clinical practice and education before and during the pandemic. Median working time in ICU was 2.5 months/year before the pandemic and 6 months/year during the pandemic, which was significantly increased (*P* < .001). While working time in the operating room was 9 months/year before the pandemic, it was 6 months/year during the pandemic and decreased significantly (*P* < .001). Working time in the algology department and anaesthesiology outpatient clinic also decreased significantly during the pandemic (both *P* < .001). Neuraxial and peripheral nerve block practices significantly decreased during the pandemic (*P* = .002 and *P* = .023, respectively). The residents worked a median of 7 night shifts per month before the pandemic and 8 night shifts per month during the pandemic, which was significantly different (*P* < .001). While the average number of beds in ICU was 14 before the pandemic, it increased significantly and reached 19.5 beds during the pandemic (*P* < .001). The education time for lecture and clinical case discussion was 2 h/week in the clinics before the pandemic; however, it decreased to 1.5 h/week during the pandemic (*P* < .001). Medical meeting attendance did not change statistically during the pandemic (*P* = .174). 

**Figure 1.**
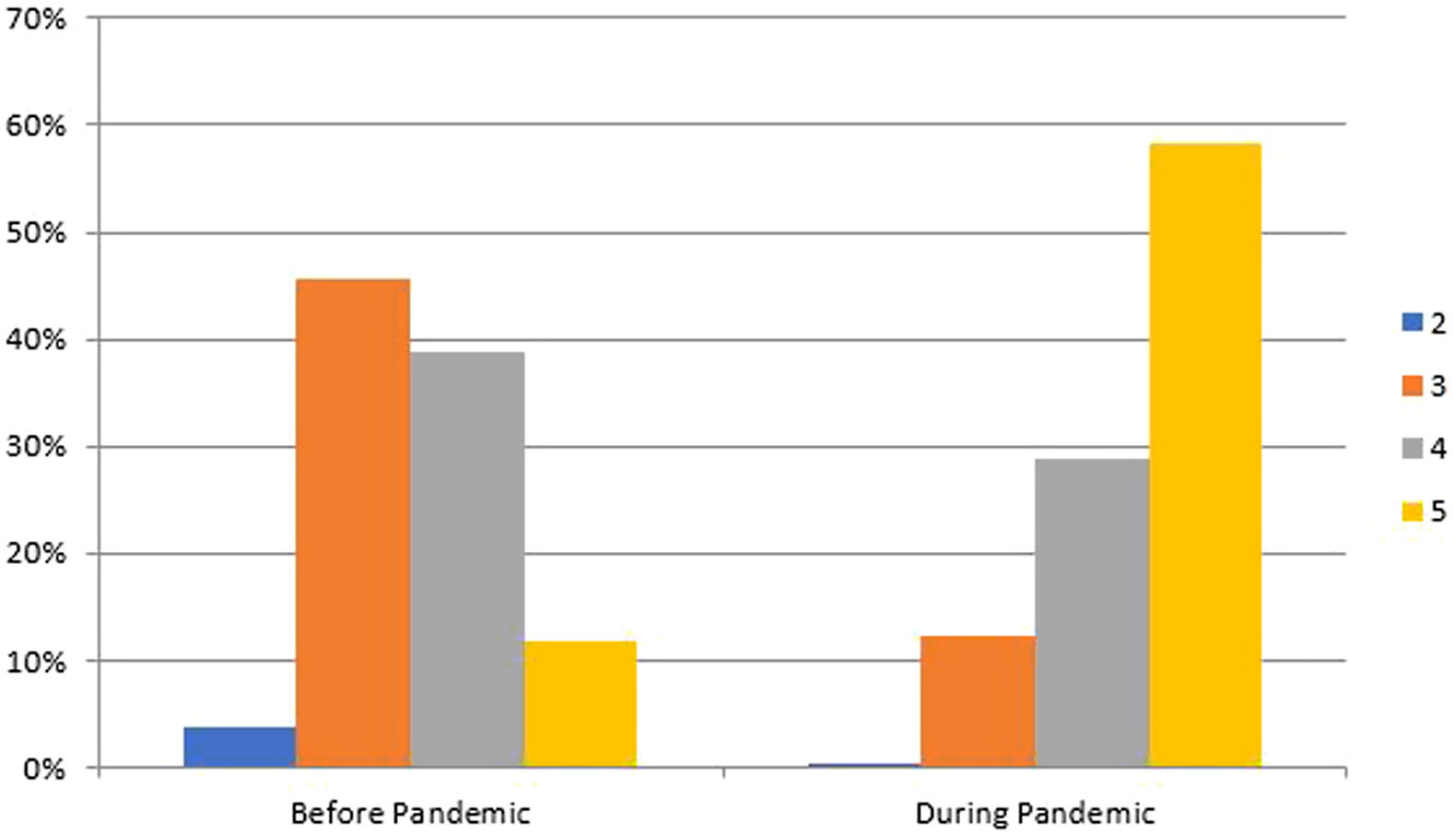
Subjective perspective on night shift intensity.

The questions asked about night shift intensity before and during the pandemic were Likert scales which were graded on a 1-5 scale with 1 representing “not intense” and 5 representing “very intense.” None of the participants reported “1” both before and during the pandemic, 45.6% of the participants reported “3” and 38.7% “4” before the pandemic. However, 58.3% of the participants reported “5” and 28.9% “4” during the pandemic (Figure 1). The difference was statistically significant (*P* < .001).

For 87.3% of the respondents, the workload increased during the pandemic, and 71.6% of the respondents reported a decrease in theoretical education and 66.7% in practical training during the pandemic (Table 3). Forty-seven of the last year residents reported that they were negatively and very negatively affected in finding a thesis topic, collecting data, and writing the thesis. 

The last question was answered by 49 of the participants. Sixteen of them stated that they had nothing to add. Most respondents stated that their workload was too high, they could not get out of ICU, they did not receive adequate training in anaesthesiology, and they were psychologically exhausted.

## Discussion

Findings from the survey showed that the working time of anaesthesiology and reanimation residents in ICU increased, and the working conditions became intense during the pandemic in Turkey. However, anaesthesiology and algology practices decreased. Anaesthesiology and reanimation residents thought that the clinical workload increased plenty much, while training decreased during the pandemic. 

Many surveys have been conducted from different countries and different specialties examining the impact of the COVID-19 pandemic on resident training.^1-3,6-9^ Previous studies showed that pandemic was related to a significant reduction in residents’ training. While the impairment was more severe in surgical specialties, it has been shown that internal specialties such as cardiology were also affected.^8^ On the other hand, it is thought that some specialties such as internal medicine, infectious diseases, and anaesthesia could use and develop their skills while managing COVID-19 patients.^9^ Since patient management in ICU is a part of the training of anaesthesiology and reanimation residents, they are well-suited to care for patients in a critical care unit. In the pandemic, anaesthesiology and reanimation residents were able to quickly adapt to intensive care environments and served as a fluid workforce. This might be an educational value for them to fill the gap that may arise because of the reduction of elective surgeries and operating room cases.^10^ This study showed a significantly increased number of months that anaesthesiology and reanimation residents worked in the ICU with an increased number of intensive care beds during the pandemic. The Medical Specialization Council organizes medical residency training in Turkey. They recommend working 12 months in ICU during the 5-year anaesthesiology and reanimation resident training.^11^ During their 5-year education, it is seen that they complete half of the time they need to work in ICU in 1 year. 

In this study, we found that the working time in the operating room significantly decreased during the pandemic. Worldwide, most elective surgeries have been canceled, only emergency surgeries, cancer surgeries, and obstetrics have been undertaken.^5^ Previous studies showed surgical cases in orthopedics, urology, and ophthalmology decreased.^3,7,9^ However, labor and delivery activity did not reduce.^6^ The limited capacity of operating rooms has reduced the opportunity for on-site training in most surgical specialties.^12^ The cancelation of elective orthopedic surgeries has also affected regional anaesthesia practices.^10^ In our study, we also found that neuraxial and peripheral nerve block performances decreased during the pandemic. The relatively decreased number of elective surgeries in our country may have contributed to this result. Rios Medina and Caicedo Salazar^13^ emphasized that a dramatic decrease occurred in the regional anaesthesia training of anaesthesia residents during the pandemic because only the well-trained physicians performed the blocks. Since we did not ask about this topic, we cannot make any comments that will contribute.

This study showed that the working times in the algology department and anaesthesiology outpatient clinic significantly decreased during the pandemic. In a study investigating the effect of the pandemic on anaesthesia and reanimation education in the province of Izmir in Turkey, 71.6% of the participants reported that their algology training was disrupted due to the pandemic.^14^ In a survey study conducted by Onat et al.^15^ with anaesthesiology and reanimation residents in Turkey in 2019, only 26% of the respondents rated the duration of pain management training as sufficient. It can be concluded that while algology training rotations were insufficient even before the pandemic, this inadequacy increased with the pandemic. Telemedicine has become a priority during the pandemic as a way to replace outpatient clinical care to protect both the patient and the provider. The use of telemedicine has been reported in ophthalmology, urology, and paediatric otorhinolaryngology.^2,3,8^ Since telemedicine is not common in Turkey and in anaesthesiology and reanimation specialty, we did not ask the participants about it. However, the use of telemedicine can be considered in the fields of algology and anaesthesiology outpatient clinics. In the study where 95% telehealth use was reported since the onset of the pandemic in the USA, 82% of the urology residents reported that they did not receive training on this subject. Patients and residents would benefit from training on the effective use of telehealth .^3^

Respondents stated that they worked a median of 7 night shifts per month before the pandemic, while they worked a median of 8 night shifts during the pandemic. In Onat et al’s^15^ study, it was concluded that they worked a mean of 7.49 ± 1.99 night shifts per month. In Turkey, when the residents have night shifts, they work in the hospital for 36 hours. In Europe, weekly working hours are limited to a maximum of 48 hours for patient safety and standard of living. In addition, a minimum of 11 hours of rest per day is required.^16^ Although there has been a significant increase in the number of night shifts during the pandemic, anaesthesiology and reanimation residents in Turkey always work a lot. Moreover, in this study, it was concluded that the intensity of night shifts increased much compared to before the pandemic.

Our results showed that the education times for lecture and clinical case discussion significantly decreased, but medical meeting attendance did not change for anaesthesiology and reanimation residents in Turkey. Face-to-face medical meetings and conferences have been postponed or canceled in Turkey as well as around the world. Online-based teaching methods have increased instead of traditional methods.^7^ Although it is thought that virtual learning cannot replace face-to-face learning,^10^ many studies have reported that it is useful and cost-effective.^17^ However, clinical and practical learning is one of the most important sides, particularly for anaesthesiology.^10^ In our study, 71.6% of the respondents reported a decrease in theoretical education and 66.7% in practical training during the pandemic. Similarly, 58% of those who participated in the study of Ince et al.^14^ reported a decrease in education and 56.8% reported that the training process was inadequate during the pandemic.

In Onat et al’s^15^ study, more than 90% of the participants reported that anaesthesiology and reanimation training process was tiring and stressful in Turkey. In our study, for 87.3% of the respondents, the workload increased during the pandemic. Actually, it can be predicted that the clinical workload is matchless for anaesthesiology and reanimation specialists and residents who provide frontline care during the COVID-19 pandemic.^4^ We would like to emphasize the increase in the workload with the result of our study. 

Sixty-three percent of the last year residents who participated in our study reported that they were negatively and very negatively affected in finding a thesis topic, collecting data, and writing the thesis. In a survey study conducted with ophthalmology residents, a 40% increase was found in research productivity. It was concluded that the decrease in clinical workload leaves time for research.^9^ On the contrary, the increased workload for anaesthesiology and reanimation residents has caused them to be unable to even finish their thesis.

There are several notable limitations of our study. First, there may have been recalling and sample bias as our data were collected via an online survey. Second, this is a cross-sectional study that represents the reality of Turkey. Third, the respondent rate is 20.8%, and therefore, it is not indicative of the entire population. In Turkey, a lot of surveys related to the pandemic have been conducted, and therefore, the willingness of the participants has decreased. 

## Conclusion

In conclusion, the results of this study showed that the pandemic had a negative impact on anaesthesiology and reanimation residency training programs in Turkey. Similar studies investigating how the pandemic affects anaesthesiology and reanimation training should be conducted. In addition, new strategies should be developed to minimize the effects on training programs in the event of a future pandemic. 

### Ethics Committee Approval:

The study was approved by the Ethical Committee of Dışkapı Yıldırım Beyazıt Education and Research Hospital, Ankara, Turkey on February 22, 2021 (105/07).

### Informed Consent:

N/A.

### Peer-review:

Externally peer-reviewed.

### Author Contributions:

Concept – A.D.; Design – B.S.U.; Supervision – A.D.; Materials – B.S.U.; Data Collection and/or Processing – B.S.U., A.A.; Analysis and/or Interpretation – B.S.U., A.A., A.D.; Literature Search – A.A.; Writing Manuscript – B.S.U.; Critical Review – A.D.

### Declaration of Interest:

The authors have no conflict of interest to declare.

### Funding:

The authors declared that this study has received no financial support.

## Figures and Tables

**Table 1.** Participant Characteristics

Age, median (range)	29 (24-49)
Sex, n (%) Female Male	119 (53.4) 104 (43.6)
Training and research hospitals, n (%) University, n (%)	65 (29.1) 158 (70.9)
COVID-19 reference hospital, n (%)	223 (100)
Year of residency, n (%) First Second Third Fourth Fifth (last)	19 (8.5) 36 (16.1) 53 (23.8) 41 (18.4) 74 (33.2)

COVID-19, coronavirus disease 19.

**Table 2.** Clinical Practice and Education Before and During the Pandemic

		*P* *
How many months a year did you work in intensive care?	BP: 2.5 (2-3) DP: 6 (4-7)	<.001
How many months a year did you work in operating room?	BP: 9 (8-10) DP: 6 (4-8)	<.001
How many months a year did you work in algology department?	BP: 1 (0-2) DP: 0 (0-1)	<.001
How many months a year did you work in anaesthesiology outpatient clinic?	BP: 1 (1-2) DP: 1 (0-2)	<.001
How many neuraxial blocks were you performing per month?	BP: 10 (5.8-23.5) DP: 10 (3-20)	.002
How many peripheral nerve blocks were you performing per month?	BP: 5 (1-10) DP: 3 (0-8)	.023
How many night shifts did you work per month?	BP: 7 (6-8) DP: 8 (6.8-8)	<.001
How many beds were there in the intensive care unit you work in?	BP: 14 (9-20) DP: 19.5 (13-26)	<.001
How many hours per week was your education time for lecture and clinical case discussion in your clinic?	BP: 2 (2-3) DP: 1.5 (1-2)	<.001
How many hours per month did you attend medical meetings, excluding clinical training?	BP: 2 (0-3) DP: 2 (0-4)	.174

Values are given as median (first quarter-third quarter).

BP, before pandemic; DP, during pandemic.

*Wilcoxon test.

**Table 3.** Subjective Perspective on the Workload and the Education

	Decreased (n, %)	Not changed (n, %)	Increased (n, %)
Workload	4 (2)	22 (10.8)	178 (87.3)
Theoretical education	146 (71.6)	41 (20.1)	17 (8.3)
Practical training	136 (66.7)	44 (21.6)	24 (11.8)

Values are given as numbers (%).
